# 22q11.2 Deletion Syndrome, Oral-Maxillo-Facial Manifestations and Cognitive Functioning: Three Illustrative Case Reports

**DOI:** 10.3390/children13030355

**Published:** 2026-02-28

**Authors:** Dario Sardella, Andrea De Giacomo, Andrea Ricci, Luisa Limongelli, Massimo Corsalini

**Affiliations:** 1 Translational Biomedicine and Neuroscience Department (DiBraiN), University of Bari “Aldo Moro”, 70124 Bari, Italy; d.sardella8@phd.uniba.it; 2 Specialisation School of Paediatric Dentistry, Department of Interdisciplinary Medicine D.I.M., University of Bari Aldo Moro, 70124 Bari, Italy

**Keywords:** 22q11.2 DS, cognitive functioning, oral manifestations

## Abstract

**Highlights:**

**What are the main findings?**

- All children with 22q11.2 deletion syndrome consistently exhibited a history of dental caries, and enamel hypomineralization was observed in two-thirds (two out of three) of the reported cases.
- In all reported cases, there was at least one pediatric neuropsychiatric comorbidity.

**What are the implications of the main findings?**

- Following an improvement in the medium- to long-term prognosis of patients with 22q11.2 DS, the implementation of early and multidisciplinary care pathways is essential.
- There is a clear need for targeted awareness and support programs on dental hygiene and mental health for individuals and families affected by 22q11.2 deletion syndrome.

**Abstract:**

**Background:** 22q11.2 deletion syndrome (22q11.2 DS) is a rare genetic syndrome characterized by high phenotypic variability, with an incidence of approximately 1:4000 live births. Most of the existing literature consists of case reports or case series, making it challenging to obtain large cohorts for data comparison and drawing generalizable conclusions. **Aim:** The aim of this article is to share the clinical experience of patients with 22q11.2 DS who were previously evaluated by Child Neuropsychiatry and underwent odontoiatric procedures at the Dental Unit of the Policlinico di Bari. **Methods:** We report three clinical cases of pediatric patients with 22q11.2 DS who were managed at the University Dental Unit of the Policlinico di Bari for dental procedures, including pre/post-treatment images and child neuropsychiatric characteristics. **Results:** Cleft lip and cleft palate are the most commonly encountered facial malformations. Enamel hypoplasia and hypomineralizations, caries, dental agenesis, and hypodontia are reported with variable frequency in almost all studies considering the oral health of these patients. Our experience is coherent with the data present at the moment. The clinical examinations showed diffuse hypomineralization and several caries, concordant with the literature. One patient received moderate sedation and another received general anesthesia; oral prophylaxis, fluoride application, pulp therapy, extractions of hopeless teeth and restorations of carious and hypomineralized teeth were performed. **Conclusions:** 22q11.2 DS is a genetic condition with a variable prognosis, but current trends show that patients often reach adulthood, primarily due to advancements in cardiology, which was previously the leading cause of mortality in these patients. From this perspective, collaboration among various medical specialties is crucial to implement holistic patient management programs and early interventions.

## 1. Introduction

The main aim of this article is to share the clinical experience of the integrated Dental-Child Neuropsychiatric management of three children affected by 22q11.2 DS in order to address the gap in integrating dental management with neuropsychiatric profiling in young patients with 22q11.2 deletion syndrome.

DiGeorge syndrome is now recognized as part of the spectrum of 22q11.2 deletion syndrome. Current nomenclature recommends using “22q11.2 deletion syndrome” when the genetic deletion is confirmed, whereas “DiGeorge syndrome” is reserved for individuals presenting the clinical phenotype without a known deletion [1]. 22q11.2 DS, also referred to as velocardiofacial syndrome (VCFS) due to its varied clinical presentations, is a syndrome of genetic etiology. The most common genetic cause is a deletion of at least 3 Mb in the q11.2 region of chromosome 22 [2]. Usually, the mutation is de novo, but some cases of autosomic dominant transmission have been reported. The incidence of the disease is equal among the sexes and approximately 1:4000 live-born infants are affected, but the incidence is actually increasing due to the rising number of affected adults having their own affected children and TREC-based newborn screening programs [3,4].

Due to the severity of the clinical phenotype associated with 22q11.2 deletion syndrome, researchers are exploring the implementation of 22q11.2 deletion testing within non-invasive prenatal screening (NIPS) protocols for high-risk pregnancies, demonstrating clinical benefit [5].

Major phenotypic features include the following [6]:

- Cardiac anomalies;
- Immune deficiency;
- Palatal defects;
- Altered social and cognitive functioning.

### 1.1. Cardiac Anomalies and Immune Deficiency

Congenital Heart Diseases (CHDs) are prevalent in approximately 1 in 100 live births and constitute the most frequently observed congenital anomalies [7]. According to one of the last meta-analyses published about this topic by Sauter et al. [8], we report the most common CHDs followed by range of estimated frequency:

Interrupted aortic arch type B (50–89%)
Truncus arteriosus (34–41%)
Tetralogy of Fallot (8–35%)
Isolated aortic arch anomaly (5–24%)

Fetal ultrasound can be one of the first prenatal screens identifying the fetus with an aortic arch anomaly but normal intracardiac anatomy, at risk for 22q11.2 DS [9].

The 22q11.2 deletion syndrome (22q11.2 DS) is a genetic syndrome historically associated with thymic absence (1% of cases) or hypoplasia (95% of cases) [10].

It is estimated that 67–80% of individuals with a 22q11.2 deletion exhibit some degree of T-cell lymphopenia (TCL) [11]; as a result, the majority of patients affected by 22q11.2 DS are more susceptible to recurrent infections such as viral respiratory infections, otitis, and sinopulmonary infections [12].

Furthermore, patients with this genetic syndrome are susceptible to developing autoimmune conditions, including idiopathic thrombocytopenic purpura (ITP), juvenile idiopathic arthritis (JIA), and, as recently documented, systemic lupus erythematosus [13,14].

Without a functional thymus, after Hematopoietic Stem Cell Transplantation (HSCT), immune reconstitution may occur via peripheral expansion of mature donor T cells. Additionally, in cases of aplasia, thymus transplantation has been proven to restore normal immune function [15].

### 1.2. Maxillo-Facial and Oral Manifestations

Children with 22q11.2 deletion syndrome generally present with a long and narrow face [16,17].

The most frequently observed anomalies include the following: hypertelorism; narrow, downslanting palpebral fissures; low-set ears with small, dysmorphic auricles; a prominent nose with a rounded tip and alar hypoplasia in younger children, evolving into a ‘tubular’ shape with a broad tip and alar hypoplasia in older children; a short philtrum; micrognathia; and an everted upper lip. Some authors have proposed a screening role of these features within the context of machine learning and facial analysis programs [18], others have used craniofacial dysmorphology as a predictor for the further development of psychosis [19].

The 22q11.2 deletion is detected in 75% of individuals with palatal anomalies [20].

A cleft lip and/or cleft palate are among the most common congenital malformations occurring in the craniofacial region, with a worldwide incidence of approximately 1 in 700 live-born infants [21].

The correlation between the presence of a cleft palate and the 22q11.2 microdeletion has been demonstrated in several pediatric population studies, with a variable frequency ranging from 0% to 14% [10], these manifestations can lead to feeding and swallowing difficulties, nasal regurgitation, speech and sleep disorders, and a predisposition to recurrent otitis that can further cause conductive hearing loss.

The prevalence of palatal clefts is higher compared to age-matched healthy controls. Isolated palatal clefts were most frequently observed (33%), followed by submucous clefts (16%) [22]. An isolated palatal anomaly rarely justifies screening for the 22q11.2 deletion; however, a palatal anomaly accompanied by additional manifestations—particularly neurocognitive dysfunction or a suggestive facial phenotype—substantially increases the likelihood of the deletion [23]. In cases where surgical intervention for these palatal defects is required, 22q11.2 DS does not seem to be associated with an increased incidence of adverse events during the postoperative period [24].

In patients with 22q11.2 DS, dental anomalies have been described, including eruption disturbances, and anomalies of number (dental agenesis, hypodontia) and shape and structure (enamel hypoplasia and hypomineralization). These disorders are significant risk factors for carious lesions.

According to a recent retrospective study conducted at the Royal Children’s Hospital in Melbourne on a cohort of 57 children with 22q11.2 deletion syndrome (22q11.2 DS), 77.2% exhibited caries, 54.4% showed developmental defects of enamel (DDE), and 12.3% presented with developmentally missing teeth [25]. The elevated incidence of caries in patients with 22q11.2 deletion syndrome (22q11.2 DS) has been attributed by some authors to qualitative differences in saliva, including elevated protein and IgA concentrations and reduced levels of electrolytes such as calcium, phosphate, and bicarbonate ions, alongside a generally acidic pH [26].

A Finnish study group retrospectively examined 45 orthopantomograms, revealing that in 17% of cases there was dental agenesis. The missing teeth were primarily mandibular incisors [22]. Data are consistent with those of following studies in which tooth agenesis was observed in 15% of participants. Enamel disturbances were present in 66% of participants and affected 26.0% of teeth. Specifically, hypomineralizations were identified in 24.0% of individuals and 18.7% of teeth, while hypoplasias were found in 8.0% of participants and 7.5% of teeth. Seventeen participants (34.0%) exhibited both types of disturbances; however, these rarely co-occurred on the same tooth, with only two teeth (0.17%) presenting both defects. Hypomineralizations were twice as prevalent in permanent teeth compared to primary teeth [17]. In another group, dental anomalies were observed, with a prevalence of hypoplastic alterations, particularly the underdevelopment of the lingual cusp of the first mandibular premolars and enamel opacities. However, further studies are necessary to obtain more confirmations [27].

In a case report, a histological examination of dental tissue revealed increased dentin calcification. According to the authors, these findings can be considered to be direct evidence of transient disturbances in calcium metabolism during tooth development [28]. We report in Table 1 the estimated frequencies of dental anomalies [29].

Due to the consistent findings in many case series and reviews, some authors suggest a potential role for enamel hypoplasia/hypomineralization as a clinical marker or diagnostic aid in the diagnosis of 22q11.2 DS [30].

### 1.3. Altered Social and Cognitive Functioning

Children with 22q11.2 deletion syndrome (22q11.2 DS) exhibit a higher incidence of delays in the acquisition of neurodevelopmental milestones [31,32], neurodevelopmental disorders [33] such as autism and ADHD, and psychotic spectrum disorders in adolescence [6]. Social cognition, which encompasses components such as the Theory of Mind (ToM), social perception, social knowledge, and emotion processing, is severely impaired in patients with 22q11.2 DS [34].

This predisposition may arise from structural or functional causes. Support for a structural explanation comes from a recent retrospective study at St. Thomas’ Hospital (London) in which magnetic resonance imaging (MRI) of fetuses with 22q11.2 DS revealed a statistically significant difference in white matter compared with healthy controls [35].

Conversely, a recent article revisited disrupted connectivity as a functional etiological hypothesis for autism spectrum disorder [36], reporting a mouse model named LgDel that replicates 22q11.2 DS manifestations. Age-specific patterns of brain dysconnectivity were observed, characterized by widespread fMRI hyperconnectivity in juvenile mice that transitioned to hippocampal hypoconnectivity during puberty [37].

22q11.2 DS is the strongest known genetic risk factor for schizophrenia [38]. Individuals with 22q11.2 deletion frequently experience positive symptoms throughout their lifespan, placing them in an ultra-high clinical risk category for psychosis [39,40].

In a recent study, the authors conducted a cross-sectional study comparing a group of patients with both schizophrenia and 22q11.2 deletion syndrome (22q11.2 DS) with a group consisting solely of patients with 22q11.2 DS. It is hypothesized that 22q11.2 DS itself, independently of psychosis, may influence self-esteem, or that reduced insight in patients with psychosis might mask the true impact of impaired functioning on self-esteem [41].

Pharmacological treatment was deemed necessary in some cases, with a therapeutic effect reported in 50% of these cases described. Olanzapine demonstrated a quantitative higher improvement rate (70%) compared to risperidone (33%) and quetiapine (40%); however, these differences were not statistically significant due to the small sample size. Adverse events, predominantly mild, occurred in 60% of cases, with extrapyramidal symptoms (25.7%) and weight gain (14.2%) being the most common [42].

The heterogeneity of clinical manifestations, in association with possible genetic mutations causing 22q11.2 DS, has been the subject of genotype–phenotype correlation in multiple studies analyzing different aspects such as language. Recently, the language profile in a population of school-aged children with 22q11.2 duplication syndrome (22q11.2 DupS) was compared with that of a group with 22q11.2 DS. A significant linguistic impairment was demonstrated in both groups compared to healthy age-matched peers. No statistically significant differences emerged between the two groups (22q11.2 DupS vs. 22q11.2 DS), but qualitatively, children with 22q11.2 DS appeared to have more difficulties, with lower scores compared to the 22q11.2 Dup group [43].

However, cognitive impairment is frequently observed in these patients, ranging from a borderline cognitive profile to mild–moderate intellectual disability [44].

A recent longitudinal study investigating cognitive aspects in patients with 22q11.2 DS demonstrated a statistically significant sex-based difference in the Full-Scale Intelligence Quotient (FSIQ) when re-evaluated approximately 20 years after the baseline assessment. It further indicates that females and the subpopulation affected by psychosis present with higher IQ variability, tending to show a decline of about 10 IQ points [45].

The utility of early and/or prenatal diagnosis lies in its capacity to adequately prepare families for the comprehensive management of a child with 22q11.2 DS. Consequently, the establishment of a robust support network is imperative to guarantee an optimal quality of life for the affected child and their broader family system [46,47].

## 2. Materials and Methods

We present three clinical cases of pediatric patients affected by this rare syndrome, who were referred to and managed at the Oral and Maxillofacial Surgery Unit of the Policlinico di Bari Hospital. Inclusion criteria for these cases were as follows: pediatric patients aged 0–14 years, and confirmation of 22q11.2 deletion through diagnostic techniques, including FISH (Fluorescent In Situ Hybridization) or Array AGH.

All patients included in the study underwent a comprehensive medical history, and clinical and standardized evaluations according to common clinical practice, complemented by radiographic investigations. Informed consent was obtained from the parents of all subjects at the time of admission to the Odontostomatologic Unit, Policlinico University Hospital of Bari.

## 3. Case 1 (Female Patient)

### 3.1. Childhood Neuropsychiatric History

Pre-perinatal Risk Factors: mother with a genetic diagnosis of 22q11.2 DS and intrauterine growth retardation (IUGR) during the last 4 weeks of pregnancy.

Child neurodevelopmental history is characterized by delayed acquisition of gross motor milestones, such as independent sitting and walking.

At the age of 13 years, the patient received a genetic diagnosis of 22q11.2 deletion syndrome via FISH. The same year, they underwent specialized evaluation at the Child Neuropsychiatry Unit at Policlinico di Bari University Hospital; at that time, the parents reported academic difficulties from preschool, and after standardized evaluation a border cognitive IQ was revealed. The diagnosis was “learning difficulties in a patient with border cognitive profile”.

### 3.2. Odontostomatologic History

**Figure 1:**
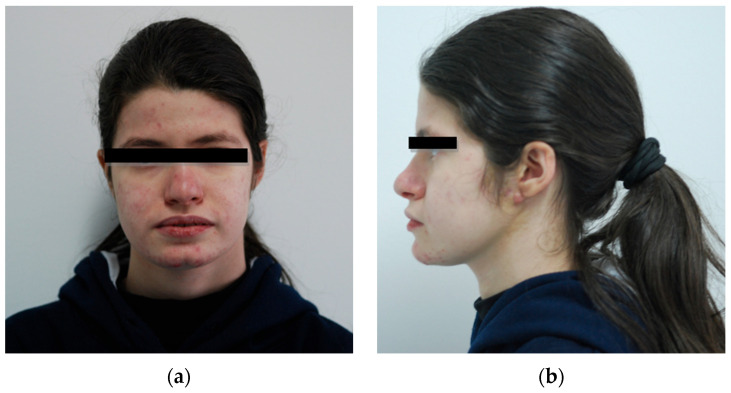
Patient n.1 frontal and lateral facial photographs: (**a**) frontal facial photograph; (**b**) left lateral facial photograph.

At 14 years old (Figure 1), the patient was referred to the Oral and Dental Diseases Division at the Policlinico di Bari University Hospital due to multiple caries and recurrent odontogenic abscesses.

**Figure 2:**
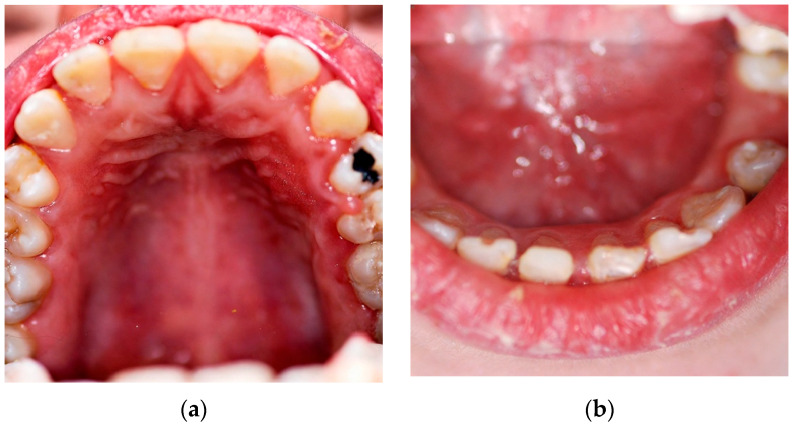
Patient n.1 superior and inferior occlusal photographs: (**a**) superior occlusal photograph; (**b**) inferior occlusal photograph showing hypomineralization lesions.

**Figure 3:**
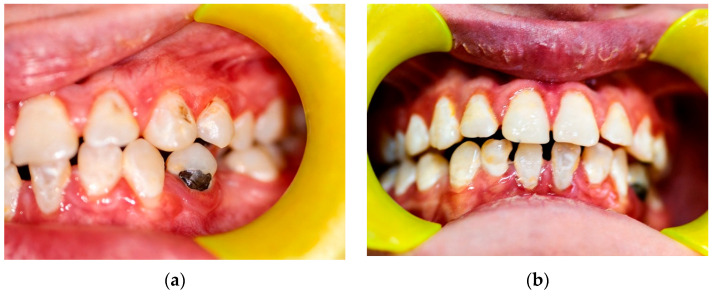
Patient n.1 frontal and left lateral mouth photographs: (**a**) left-lateral mouth photograph: overbite within normal limits, presence of diastemas in both the maxillary and mandibular arches, cervical carious lesions; (**b**) frontal mouth photograph shows marginal gingivitis and enamel hypomineralizations.

**Figure 4:**
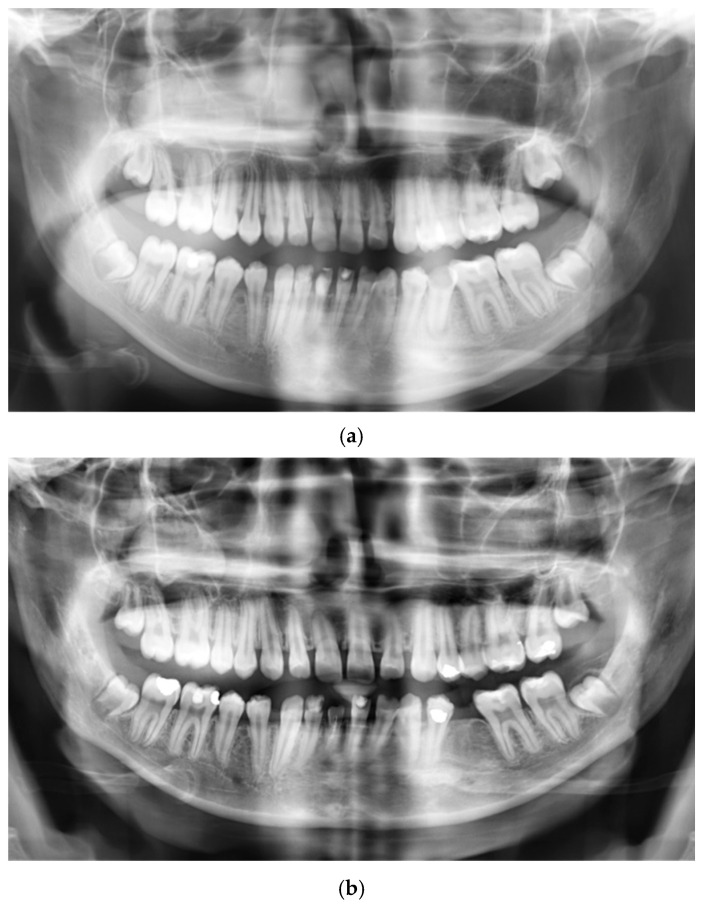
Patient n.1 pre- and post-treatment panoramic radiograph: (**a**) pre-treatment panoramic radiograph; (**b**) post-treatment panoramic radiograph shows conservative therapies, the root canal therapy of 4.1, the avulsion of 3.5 affected by periapical inflammatory disease.

Intraoral examination revealed diffuse marginal gingivitis, diffuse hypomineralizations across both dental arches, multiple dental caries, and insufficient oral hygiene within the permanent dentition (Figure 2b and Figure 3a,b). DMFT (Decayed, Missing, Filled Teeth) = 13 (D = 9, M = 0, D = 4). The following treatments were performed under moderate sedation:

- Oral hygiene instruction and prophylaxis;
- Fluoride varnish application;
- Endodontic treatment of 4.1;
- Conservative treatments for hypomineralization and carious lesions of 1.4, 2.4, 2.7, 3.4, 4.4, 4.6, 4.7;
- Avulsion of severely compromised dental element (3.5) affected by periapical inflammatory disease (Figure 4b).

During the procedure, cefazolin 1 g IV was administered as intraoperative prophylaxis. The patient was dismissed with prophylactic antibiotic therapy, amoxicillin–clavulanic acid 600/150 mg/day, divided into three daily doses, was used. Paracetamol 500 mg suppositories were prescribed as needed.

## 4. Case 2 (Male Patient)

### 4.1. Childhood Neuropsychiatric History

Pre-perinatal risk factors: patient’s mother and sister both have a genetic diagnosis of 22q11.2 deletion syndrome.

At 3 years of age, the patient underwent a child neuropsychiatric evaluation at the Child Neuropsychiatry Unit of Policlinico of Bari due to delayed language acquisition. This evaluation concluded with the diagnosis of ‘global developmental delay (case under observation)’ according to DSM-5 criteria. During this initial assessment, standardized testing could not be administered due to extremely labile attention levels, leading to the discontinuation of the Leiter-3 non-verbal cognitive test. Clinical observation revealed marked motor restlessness, severely underdeveloped verbal production, and verbal comprehension limited to simple commands. School attendance with educational support, speech therapy, and psychomotor therapy, along with genetic counseling, were recommended.

At the age of 5, genetic diagnosis via FISH confirmed 22q11.2 DS, and a re-evaluation at the Child Neuropsychiatry Unit concluded with a ‘delay in the acquisition of academic learning in patient with borderline cognitive endowment and 22q11.2 deletion syndrome.’

### 4.2. Odontostomatologic History

**Figure 5:**
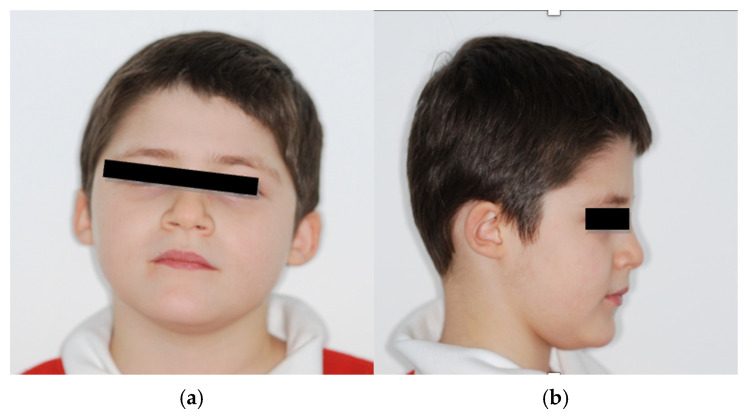
Patient n.2 frontal and lateral facial photographs: (**a**) frontal facial photograph; (**b**) right-lateral facial photograph: concave profile, lips retruded relative to the esthetic (E-) line.

**Figure 6:**
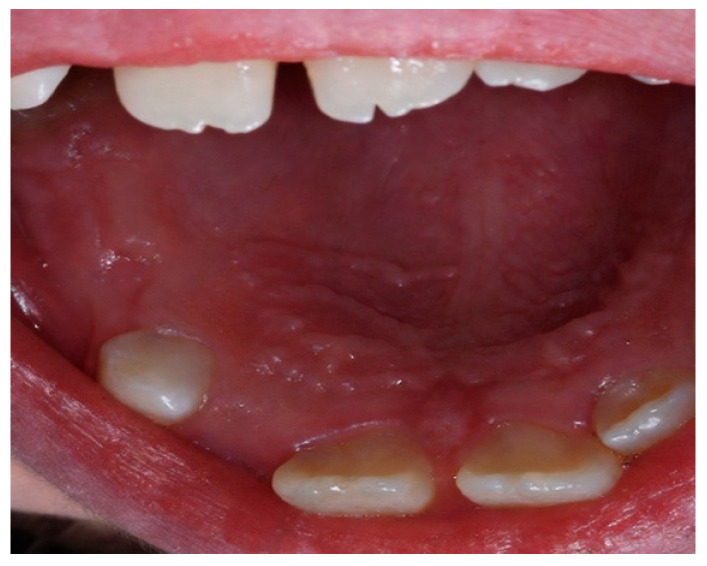
Patient n.2 superior occlusal photographs point out enamel hypoplasia.

**Figure 7:**
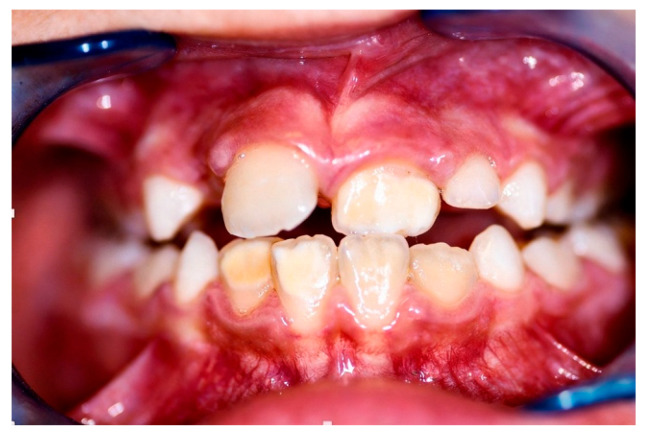
Patient n.2 frontal mouth photograph shows bilateral maxillary constriction with bilateral posterior crossbite and tooth 21 in crossbite with tooth 31.

**Figure 8:**
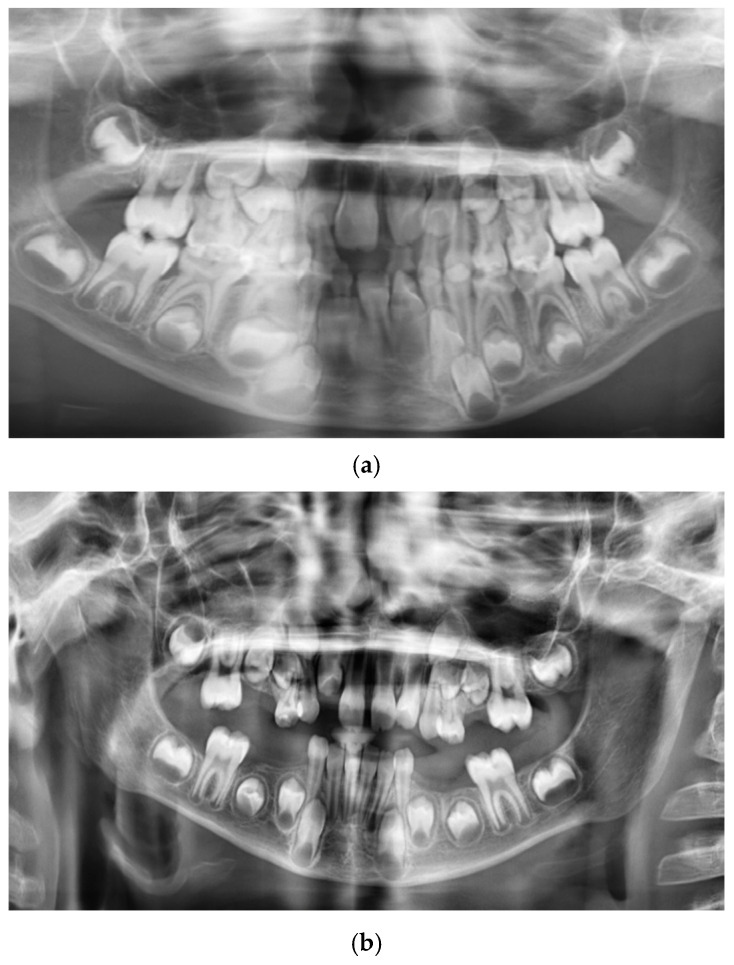
Patient n.2 pre- and post-treatment panoramic radiograph: (**a**) pre-treatment panoramic radiograph; (**b**) post-treatment panoramic radiograph.

The patient presented to the Odontostomatologic Unit of Policlinico di Bari University Hospital at the age of 8 (Figure 5) due to multiple caries and recurrent odontogenic abscesses, predominantly in the left hemimandible. Intraoral examination revealed diffuse marginal gingivitis, transverse maxillary hypoplasia of the upper arch, widespread hypomineralizations and hypoplastic defects in both dental arches, multiple caries, and insufficient oral hygiene, in mixed dentition (Figure 6, Figure 7 and Figure 8a). DMFT = 11 (D = 11, M = 0, F = 0). The following treatments were performed under general anesthesia:

- Oral hygiene instruction and prophylaxis;
- Fluoride varnish application;
- Avulsion of severely compromised dental elements 5.2, 5.4, 5.5, 6.1, 6.5, 7.4, 7.5, 8.4, 8.5 Figure 8b;
- Restorative therapies for hypomineralization defects and carious lesions (1.6, 4.6) (Figure 8b).

During the procedure, cefazolin 1 g IV was administered as intraoperative prophylaxis. Patient was dismissed with prophylactic antibiotic therapy, amoxicillin–clavulanic acid 600/150 mg/day, divided into three daily doses, was used. Paracetamol 500 mg suppositories were prescribed as needed.

## 5. Case 3 (Male Patient)

### 5.1. Childhood Neuropsychiatric History

Pre-perinatal risk factors: born full-term following dystocic delivery.

At 3 years of age, the patient underwent the first Child Neuropsychiatric evaluation due to language difficulties, which led to the diagnosis of “language delay in patient with rhinolalia”. Consequently, the patient initiated 5 years of speech therapy with clinical benefit.

At 5 years of age, a diagnosis of an “approximately 3.15 Mb deletion in the 22q11.21 chromosomal region, de novo onset” was made using an Array Genomic Hybridization (AGH) methodology.

At the age of 9, a new Child Neuropsychiatric evaluation was performed. The administration of the verbal cognitive test WISC-IV (Wechsler Intelligence Scale for Children—Fourth Edition) revealed a total IQ (Intelligence Quotient) of 71, indicative of a mild intellectual disability. Upon observation, linguistic abilities were found to be commensurate with the chronological age; however, performance anxiety and academic learning difficulties were noted. Consequently, a diagnosis of an “unspecified learning disorder in a patient with mild intellectual disability and 22q11.2 deletion syndrome” was made according to DSM-5 criteria, and school attendance with compensatory and dispensatory aids was recommended.

At 11 years of age, a further specialist evaluation administered the same WISC-IV cognitive test, yielding the following results: IQ 46. The diagnosis was updated to “moderate intellectual disability in a patient with 22q11.2 deletion syndrome,” and school attendance with educational support was recommended.

### 5.2. Odontostomatologic History

**Figure 9:**
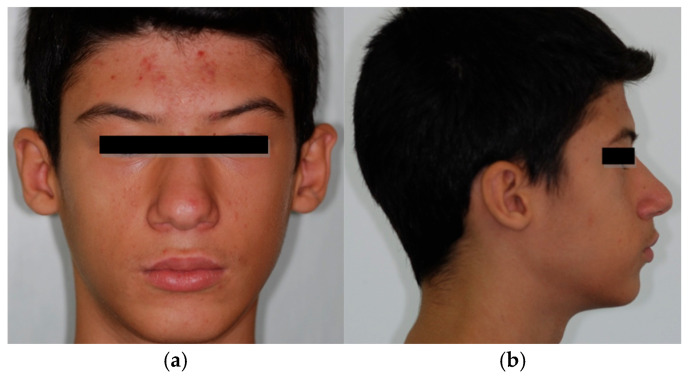
Patient n.3 frontal and lateral facial photographs: (**a**) frontal facial photograph; (**b**) right lateral facial photograph: concave profile; adequate lip display relative to the esthetic (E-) line.

**Figure 10:**
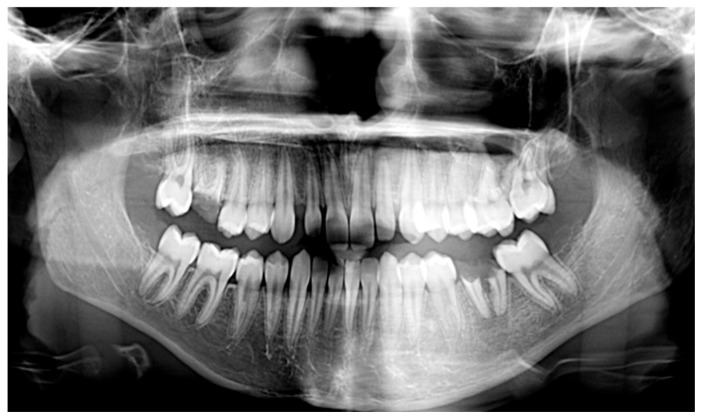
Patient n.3: pre-treatment panoramic radiograph shows rarefying osteitis associated with destructive caries of 3.6.

At 13 years of age (Figure 9), the patient was referred to the Dental and Oral Surgery Unit of Policlinico di Bari for destructive crown-root caries on teeth 1.6 and 3.6, and recurrent odontogenic abscesses predominantly affecting the left hemimandible, Figure 10. DMFT = 2 (D = 2, M = 0, F = 0). For this patient, avulsion of teeth 1.6 and 3.6 and conservative treatments under deep sedation were planned; however, the patient did not attend on the day of the procedure.

## 6. Discussion

The three patients included in these case reports showed characteristics similar to those reported in the literature.

All three children had a neurodevelopmental history characterized by delays [6] in acquiring developmental milestones: in two out of 3 three cases, the delay was in language acquisition [42], and in one out of three cases, it was in gross motor development [39].

Regarding cognitive functioning, as measured by standardized scales, in one out of three cases (33%) an IQ within the normal range was observed, but this was at the lower end of the normal limits; meanwhile, in two out of three cases (66%), a diagnosis of mild-to-moderate intellectual disability was formulated [43]. Regardless of intellectual functioning, a higher IQ is generally associated with better social cognition performance [34].

Contrary to findings reported in a recent publication, our study detected cognitive decline in only a single male patient [44]. Although at the time of the most recent pediatric neuropsychiatric assessment case 3 did not exhibit behaviors attributable to schizophrenia spectrum disorders, the cognitive decline (−35 IQ points over 2 years) may be interpreted as a strong indicator of an increased risk for developing a psychotic illness [40]. Furthermore, in a cohort of 412 children with 22q11.2 deletion syndrome, the minimum age of onset of psychotic disorders was 12 years, an age compatible with that of the patient at the time of the last assessment. The patient failed to attend on the day of the procedure; this circumstance underscores the need for close follow-up to identify at-risk mental states early and to initiate timely psychopharmacological intervention when indicated.

None of the described cases exhibited behaviors attributable to autism spectrum traits.

All children received a form of educational support corresponding to their needs or level of compromission.

All patients exhibited caries of varying severity [26], and in two out of three cases, hypomineralization was observed [30]. For these reasons, two out of three patients received fluoride varnish application to enhance remineralization and reduce the risk of future caries.

The patient compliance level and the invasiveness of the dental extraction procedure are the primary determinants of the type of sedation selected.

Case 1 is a girl with a borderline IQ but one within the normal range; levels of agitation and anxiety were low when family members were present. After anesthesiologist evaluation, we decided to proceed with moderate sedation [48] during the endodontic procedure.

Case 2 is a boy diagnosed with global developmental delay, so severely impaired that completion of standardized testing was not possible. He was unable to follow simple commands or cooperate with healthcare personnel; during the preoperative anesthesiologic consultation, he was classified as Mallampati score II. The dental treatment plan included the extraction of primary teeth with a questionable prognosis under general anesthesia, given the patient’s high risk of new caries due to poor compliance, low adherence to prior preventive programs, restlessness, and inadequate cooperation for both the surgical procedure and follow-up conservative treatments.

Regarding susceptibility to inflammation, the literature indicates that patients with 22q11.2 deletion syndrome exhibit impaired T-lymphocyte maturation, which predisposes them to chronic inflammatory conditions such as gingivitis and periodontitis. We found periapical inflammation in case 1 (33%). Marginal gingivitis was found in two out of three cases (66%) [11].

All patients received comprehensive oral care, and parents were provided with specialized instruction on appropriate oral hygiene.

The major limitations of this study are the lack of longitudinal follow-up and the sample size, which includes only three pediatric patients. The small sample size, attributable to the rarity of 22q11.2 deletion syndrome, precludes generalizable conclusions; nevertheless, the article aims to share the accumulated clinical experience of the integrated management.

## 7. Practical Implications

Practical points for pediatric dentists and orthodontists:

- Early interdisciplinary screening: refer patients with DiGeorge phenotype or confirmed 22q11.2 DS to the pediatrician, immunologist, and child neuropsychiatrist;
- Close surveillance and intensified prevention: early detection programs, personalized home hygiene and regular fluoride varnish applications;
- Management of hypomineralizations and caries: early conservative treatments, of sealants, and protective restorative materials to prevent endodontic complications;
- Moderate/deep sedation chosen based on compliance and experience: decision based on cooperation, anxiety levels and preoperative anesthesiologic assessment;
- Caregiver education and long-term follow-up: give simple instructions, involve school/therapists, and adapt plans according to the patient’s overall development;

## 8. Conclusions

We report three pediatric cases with molecularly confirmed 22q11.2 DS, focusing on their neuropsychiatric and dentofacial histories. Historically, this genetic syndrome carried a poor long-term prognosis largely due to cardiac complications; however, improved care has increased survival, and many patients now reach adulthood and may form families. After recent advances in early diagnosis and management, multidisciplinary collaboration among relevant specialties is essential [45]. Such collaboration should enable early identification and, together with families, the establishment of coordinated medical surveillance and habilitative/rehabilitative programs to improve the short- and long-term outcomes and quality of life for individuals with 22q11.2 DS [46].

## Figures and Tables

**Table 1:** Estimated frequencies of dental anomalies according to Funato.

Dental Anomaly	Estimated Frequency
Enamel Hypomineralization of Permanent Teeth	41%
Enamel Hypomineralization of Primary Teeth	39%
Hypoplasia of Primary Teeth	32%
Tooth Agenesis	15%
Hypoplasia of Permanent Teeth	10%

## Data Availability

The data presented in this study are available on request from the corresponding author due to ethical reasons.

## References

[1] Chamarthi V.S., Lackey A.E., Chamarthi S. (2025). DiGeorge Syndrome. StatPearls.

[2] McDonald-McGinn D.M., Sullivan K.E., Marino B., Philip N., Swillen A., Vorstman J.A.S., Zackai E.H., Emanuel B.S., Vermeesch J.R., Morrow B.E. (2015). 22q11.2 deletion syndrome. Nat. Rev. Dis. Prim..

[3] McDonald-McGinn D.M., Sullivan K.E. (2011). Chromosome 22q11.2 deletion syndrome (DiGeorge syndrome/velocardiofacial syndrome). Medicine.

[4] Kwan A., Abraham R.S., Currier R., Brower A., Andruszewski K., Abbott J.K., Baker M., Ballow M., Bartoshesky L.E., Bonagura V.R. (2014). Newborn screening for severe combined immunodeficiency in 11 screening programs in the United States. JAMA.

[5] Cong X., Hu L., Pei Y., Zhong J., Song J., Wen L., Zhang T., Liu Y., Liu W. (2025). Evaluating the effectiveness of routine noninvasive prenatal screening for CNVs in 22q11.2 region in a cohort of 38,495 pregnancies. Sci Rep..

[6] Cortés-Martín J., Peñuela N.L., Sánchez-García J.C., Montiel-Troya M., Díaz-Rodríguez L., Rodríguez-Blanque R. (2022). Deletion Syndrome 22q11.2: A Systematic Review. Children.

[7] Fahed A.C., Gelb B.D., Seidman J.G., Seidman C.E. (2013). Genetics of congenital heart disease: The glass half empty. Circ. Res..

[8] Sauter C., Hofbeck M., Franz P., Kettenstock L., Steger K., Linhardt M., Reiter A., Störk S., Romanos M., Radtke F. (2025). Congenital heart disease in 22q11.2 deletion syndrome: A meta-analysis and systematic review of the literature. J. Med. Genet..

[9] Freud L.R., Wapner R., McDonald-McGinn D.M. (2024). Prenatal detection of 22q11.2 deletion syndrome and congenital heart disease. Am. J. Obstet. Gynecol..

[10] Cárdenas-Nieto D., Forero-Castro M., Esteban-Pérez C., Martínez-Lozano J., Briceño-Balcázar I. (2020). The 22q11.2 Microdeletion in Pediatric Patients with Cleft Lip, Palate, or Both and Congenital Heart Disease: A Systematic Review. J. Pediatr. Genet..

[11] Toka O., Karl M., Dittrich S., Holst S., Holst A. (2010). Dental aspects in patients with DiGeorge syndrome. Quintessence Int..

[12] Gennery A.R. (2012). Immunological aspects of 22q11.2 deletion syndrome. Cell Mol. Life Sci..

[13] Di Cesare S., Puliafito P., Ariganello P., Marcovecchio G.E., Mandolesi M., Capolino R., Digilio M.C., Aiuti A., Rossi P., Cancrini C. (2015). Autoimmunity and regulatory T cells in 22q11.2 deletion syndrome patients. Pediatr. Allergy Immunol..

[14] Sun C., Han P., Yan J. (2024). Systemic lupus erythematosus in a patient with 22q11.2 deletion syndrome: A case report and review of the literature. Cent. Eur. J. Immunol..

[15] Morsheimer M., Brown Whitehorn T.F., Heimall J., Sullivan K.E. (2017). The immune deficiency of chromosome 22q11.2 deletion syndrome. Am. J. Med. Genet. A..

[16] Perez E., Sullivan K.E. (2002). Chromosome 22q11.2 deletion syndrome (DiGeorge and velocardiofacial syndromes). Curr. Opin. Pediatr..

[17] Nordgarden H., Lima K., Skogedal N., Følling I., Storhaug K., Abrahamsen T.G. (2012). Dental developmental disturbances in 50 individuals with the 22q11.2 deletion syndrome; relation to medical conditions?. Acta Odontol. Scand..

[18] Wentzel C., Fernström M., Ohrner Y., Annerén G., Thuresson A.C. (2008). Clinical variability of the 22q11.2 duplication syndrome. Eur. J. Med. Genet..

[19] Roalf D.R., McDonald-McGinn D.M., Jee J., Krall M., Crowley T.B., Moberg P.J., Kohler C., Calkins M.E., Crow A.J., Fleischer N. (2024). Computer-vision analysis of craniofacial dysmorphology in 22q11.2 deletion syndrome and psychosis spectrum disorders. J. Neurodev. Disord..

[20] (2008). velo-cardio-facial syndrome: 30 Years of study. Dev. Disabil. Res. Rev..

[21] Jackson O., Crowley T.B., Sharkus R., Smith R., Jeong S., Solot C., McDonald-Mcginn D. (2019). Palatal evaluation and treatment in 22q11.2 deletion syndrome. Am. J. Med. Genet. A.

[22] Heliövaara A., Rantanen I., Arte S. (2011). Dental development and tooth agenesis in children with velocardiofacial syndrome. Int. J. Paediatr. Dent..

[23] Tang M.Y.P., Tsui S.Y.B., Chao S.Y.N., Chan K.W.E., Lee K.H. (2025). 22q11.2 deletion syndrome in children with palatal anomaly: Who and when to test?. Singap. Med J..

[24] Bergman H.J., Asti L., Kirschner R.E. (2025). An Assessment of Adverse Events in Patients with 22q11.2 Deletion Syndrome Undergoing Palatoplasty: An Analysis of the NSQIP Pediatric Database. Cleft Palate Craniofac. J..

[25] Wong D.H., Rajan S., Hallett K.B., Manton D.J. (2021). Medical and dental characteristics of children with chromosome 22q11.2 deletion syndrome at the Royal Children’s Hospital, Melbourne. Int. J. Paediatr. Dent..

[26] Klingberg G., Lingström P., Oskarsdóttir S., Friman V., Bohman E., Carlén A. (2007). Caries-related saliva properties in individuals with 22q11 deletion syndrome. Oral Surg. Oral Med. Oral Pathol. Oral Radiol. Endod..

[27] da Silva Dalben G., Richieri-Costa A., de Assis Taveira L.A. (2008). Tooth abnormalities and soft tissue changes in patients with velocardiofacial syndrome. Oral Surg. Oral Med. Oral Pathol. Oral Radiol. Endod..

[28] Fukui N., Amano A., Akiyama S., Daikoku H., Wakisaka S., Morisaki I. (2000). Oral findings in DiGeorge syndrome: Clinical features and histologic study of primary teeth. Oral Surg. Oral Med. Oral Pathol. Oral Radiol. Endod..

[29] Funato N. (2022). Craniofacial Phenotypes and Genetics of DiGeorge Syndrome. J. Dev. Biol..

[30] Gupta A., Singh R., Wander A. (2024). Enamel hypoplasia: A potential diagnostic aid in DiGeorge syndrome. BMJ Case Rep..

[31] Tang K.L., Antshel K.M., Fremont W.P., Kates W.R. (2015). Behavioral and Psychiatric Phenotypes in 22q11.2 Deletion Syndrome. J. Dev. Behav. Pediatr..

[32] Swillen A., Moss E., Duijff S. (2018). Neurodevelopmental outcome in 22q11.2 deletion syndrome and management. Am. J. Med. Genet. A.

[33] Shriberg L.D., Strand E.A., Jakielski K.J., Mabie H.L. (2019). Estimates of the prevalence of speech and motor speech disorders in persons with complex neurodevelopmental disorders. Clin. Linguist. Phon..

[34] Jalal R., Nair A., Lin A., Eckfeld A., Kushan L., Zinberg J., Karlsgodt K.H., Cannon T.D., Bearden C.E. (2021). Social cognition in 22q11.2 deletion syndrome and idiopathic developmental neuropsychiatric disorders. J. Neurodev. Disord..

[35] Cromb D., Finck T., Bonthrone A.F., Uus A., Van Poppel M., Steinweg J., Lloyd D.F., Pushparajah K., Razavi R., Counsell S.J. (2025). An exploratory fetal MRI study examining the impact of 22q11.2 microdeletion syndrome on early brain growth. J. Neurodev. Disord..

[36] Sanders A.F.P., Hobbs D.A., Knaus T.A., Beaton E.A. (2023). Structural Connectivity and Emotion Recognition Impairment in Children and Adolescents with Chromosome 22q11.2 Deletion Syndrome. J. Autism Dev. Disord..

[37] Alvino F.G., Gini S., Minetti A., Pagani M., Sastre-Yagüe D., Barsotti N., De Guzman E., Schleifer C., Stuefer A., Kushan L. (2025). Synaptic-dependent developmental dysconnectivity in 22q11.2 deletion syndrome. Sci. Adv..

[38] Van L., Boot E., Bassett A.S. (2017). Update on the 22q11.2 deletion syndrome and its relevance to schizophrenia. Curr. Opin. Psychiatry.

[39] Jalbrzikowski M. (2021). Neuroimaging Phenotypes Associated with Risk and Resilience for Psychosis and Autism Spectrum Disorders in 22q11.2 Microdeletion Syndrome. Biol. Psychiatry Cogn. Neurosci. Neuroimaging.

[40] Vorstman J.A., Breetvelt E.J., Duijff S.N., Eliez S., Schneider M., Jalbrzikowski M., Armando M., Vicari S., Shashi V., Hooper S.R. (2015). Cognitive decline preceding the onset of psychosis in patients with 22q11.2 deletion syndrome. JAMA Psychiatry.

[41] Accinni T., Kotzalidis G.D., Irelli E.C., Pasquini M., Buzzanca A. (2025). Self-Esteem and Psychopathology Differentially Relate to Real-Life and Social Functioning in People with 22q11.2 Deletion Syndrome. Int. J. Dev. Neurosci..

[42] Mosheva M., Korotkin L., Gur R.E., Weizman A., Gothelf D. (2020). Effectiveness and side effects of psychopharmacotherapy in individuals with 22q11.2 deletion syndrome with comorbid psychiatric disorders: A systematic review. Eur. Child Adolesc. Psychiatry.

[43] Verbesselt J., Solot C.B., Van Den Heuvel E., Crowley T.B., Giunta V., Breckpot J., McDonald-McGinn D.M., Zink I., Swillen A. (2023). Language Profiles of School-Aged Children with 22q11.2 Copy Number Variants. Genes.

[44] Bayat M., Bayat A. (2022). Neurological manifestation of 22q11.2 deletion syndrome. Neurol. Sci..

[45] Wallin L., Gillberg C., Knutsson J., Fernell E., Gillberg I.C., Billstedt E. (2025). 22q11.2 Deletion Syndrome: Cognitive, Visuomotor, and Adaptive Functioning Followed Longitudinally. Brain Behav..

[46] Walkowiak D., Domaradzki J. (2025). The impact of 22q11.2 deletion syndrome on caregivers: Assessing quality of life and burden. Orphanet J. Rare Dis..

[47] Khan A., Mazeri N., Gadancheva V., Mulcahy W., Kelleher S., McNicholas F. (2025). Evaluation of a newly introduced parenting support programme for families of children with 22q11.2 Deletion Syndrome. Ir. Med. J..

[48] AlQarni M.A., Alharbi A., Merdad L. (2018). Dental management of a patient with 22q11.2 deletion syndrome (22q11.2DS). BMJ Case Rep..

